# *Yersinia pestis* plasminogen activator protease is regulated by the PhoP/PhoQ two-component system

**DOI:** 10.1128/jb.00357-25

**Published:** 2025-12-23

**Authors:** Kenneth T. Appell, Wanfeng Guo, Madeleine Scott, Jon S. Blevins, Roger D. Pechous

**Affiliations:** 1 Department of Microbiology and Immunology, University of Arkansas for Medical Sciences, Little Rock, Arkansas, USA; 2 Department of Microbiology and Immunology, East Carolina University 3627, Greenville, North Carolina, USA; National Institutes of Health, Bethesda, Maryland, USA

**Keywords:** omptin proteases, PhoP/PhoQ, two-component regulatory systems, Pla, plasminogen activator protease, plague, *Yersinia pestis*, *Yersinia*

## Abstract

*Yersinia pestis* is a gram-negative bacterium and the causative agent of plague. The *Y. pestis* virulence factor plasminogen activator protease (Pla) is an outer membrane aspartic protease that facilitates the dissemination of bacteria from the site of inoculation to deeper tissue during bubonic plague. During pneumonic plague, Pla acts as an adhesin, which contributes to the suppression of early innate immune responses in the lungs, and as a protease that aids in resisting bacterial killing by neutrophils. Two-component regulatory systems (TCSs) are involved in bacterial adaptation to environmental stressors such as changes in pH, changes in ion concentrations, and the presence of cationic antimicrobial peptides. TCSs consist of a membrane-bound sensor kinase that detects environmental stressors and activates a response regulator to coordinately alter gene expression. The PhoP/PhoQ TCS regulates virulence factors and known Pla homologs in a variety of gram-negative pathogenic bacteria including *Escherichia coli* and *Salmonella* species. In the work described here, we evaluate whether *pla* is regulated by PhoP/PhoQ in *Y. pestis*. We identify a putative PhoP-binding site within the −10 box and the +1 transcription start site of *pla* that is bound by recombinant PhoP. Surprisingly, we show that the expression of *pla* is suppressed by PhoP/PhoQ under a variety of physiologically relevant PhoP/PhoQ-inducing conditions that are expected to be encountered during infection. This work demonstrates the regulation of an essential *Y. pestis* virulence factor by the PhoP/PhoQ TCS for the first time and highlights the importance of tightly regulating virulence factors that function as proteases.

**IMPORTANCE:**

*Yersinia pestis* causes plague, a highly lethal infection that results from inoculation via an infected flea (bubonic plague) or inhalation of contaminated respiratory droplets via person-to-person transmission (pneumonic plague). The plasminogen activator protease (Pla) is a critical *Y. pestis* virulence factor that is essential to the progression of infection via either route of inoculation. In this work, we show for the first time that the well-established two-component regulatory system PhoP/PhoQ regulates the expression of *pla*. Under conditions found during mammalian infection, PhoP/PhoQ suppresses *pla* expression, presumably to limit aberrant cleavage of Pla substrates during the critical early stages of infection. These results show interaction between two key virulence loci for the first time, and shed light on the regulation of a critical *Y. pestis* virulence determinant.

## INTRODUCTION

*Yersinia pestis* is a gram-negative coccobacillus bacterium and the causative agent of bubonic, septicemic, and pneumonic plague (1). *Y. pestis* is categorized as a Tier 1 select agent by the Centers for Disease Control and Prevention due to the high risk of deliberate misuse and potential to cause mass casualties (2). The life cycle of *Y. pestis* is complex and involves multiple hosts, temperatures, and modes of transmission (1). Natural reservoirs include wild rodents infected with *Y. pestis*, and infection is perpetuated by transmission of colonized fleas feeding on susceptible hosts (1). Humans acquire infection through bites from fleas that have previously fed on an infected host (1). Circumvention of host defenses and dissemination from the bite site and into the blood leads to septicemic plague (1). Further dissemination and colonization of the pulmonary compartment manifests as secondary pneumonic plague (3). Primary pneumonic plague is the most lethal manifestation of the disease and results from inhalation of contaminated respiratory droplets during person-to-person transmission of infection (4). *Y. pestis* harbors multiple plasmids that are essential to its virulence within a mammalian host. pPCP1 and pCD1 carry the genes encoding the plasminogen activator protease (Pla) and the Ysc type 3 secretion system (T3SS), respectively (1). Pla is an outer membrane protease encoded by the *pla* gene found on the 9.5 kb pPCP1 plasmid. pPCP1 was acquired by *Y. pestis* during the evolution from its most recent ancestor and enteric pathogen, *Yersinia pseudotuberculosis*, and Pla is a critical virulence factor essential for the progression of bubonic and pneumonic plague (1). During bubonic plague, the dissolution of fibrin clots and the proteolytic cleavage of other Pla targets are required for the dissemination of *Y. pestis* from the dermal inoculation site to the bloodstream, but Pla is not required for bacterial growth and survival at the site of infection (5). In contrast, during pneumonic plague, Pla is required for bacterial growth in the lungs, but it is not required for dissemination to other tissues (6–8).

Pla is classified as an omptin family protease sharing homology with several other enterobacterial proteases, including PgtE (*Salmonella*), OmpT and OmpP (*Escherichia coli*), and CroP (*Citrobacter rodentium)* (9–12). All of these Pla homologs are positively regulated by the PhoP/PhoQ two-component regulatory system (TCS) (10–12). The PhoP/PhoQ TCS consists of an inner membrane environmental sensor kinase, PhoQ, and a cytoplasmic transcriptional regulator, PhoP (13). When bacteria encounter environmental signals, such as low Mg^2+^, low pH, or cationic antimicrobial peptides (CAMPs), PhoQ undergoes autophosphorylation of critical cytoplasmic residues and subsequently phosphorylates PhoP (14–16). In *Y. pestis*, active PhoP binds to DNA containing specific heptameric repeats and regulates the expression of target genes across the genome (17). The PhoP/PhoQ TCS has been extensively characterized in *Salmonella* and *E. coli*, and in *Y. pestis* PhoP is thought to regulate up to 2% of the genome including genes involved in stress responses, metabolism, ion transport, and lipopolysaccharide modification (17–20). Additionally, targets may exist on pCD1 and pPCP1, implicating PhoP/PhoQ in *Y. pestis* virulence (17). Most *Y. pestis* PhoP-regulated genes are thought to be activated, but several genes are repressed by PhoP binding the promoter region near the transcription start site (21). One major target of PhoP is the cAMP receptor protein (Crp), a known activator of *pla* transcription and global regulator of genes involved in carbon metabolism and biofilm formation (22). Due to the known regulation of omptin family proteases by PhoPQ and the lack of published mechanisms for the regulation of *pla*, we sought to determine whether the PhoP/PhoQ TCS regulates *pla* expression.

In this work, we identify a putative PhoP-binding box within the core promoter element that overlaps with the transcriptional start site of *pla*. We show that recombinant PhoP binds this sequence, and altering base pairs within the binding box sequence reduces binding affinity. Finally, we show that *pla* is largely repressed upon PhoP/PhoQ activation by various known inducing conditions, and this repression is partially alleviated upon activation of Crp-mediated regulation *in vitro*. This finding may have implications for the dynamic regulation of Pla during the progression of pneumonic plague. In summary, we demonstrate the regulation of the essential *Y. pestis* virulence factor Pla by the PhoP/PhoQ TCS for the first time, and highlight the importance of tightly regulating virulence factors that function as proteases.

## RESULTS

### Identification of a putative PhoP-binding box within the core *pla* promoter

**Fig 1:**
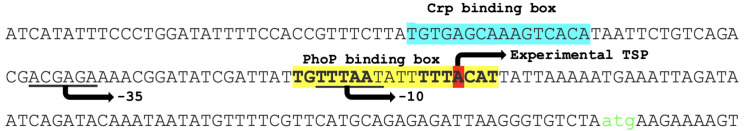
Identification of a putative PhoP-binding box with the core *pla* promoter. Sequence corresponding to a putative PhoP-binding box (highlighted in yellow) is located upstream of the *pla* coding, where PhoP overlaps with the −10 box and the experimentally defined transcription start site (TSP highlighted in red) (22). The Crp-binding box is highlighted in blue. The *pla* start codon is indicated in green lowercase.

Several omptin family proteases (e.g., PgtE, OmpP, OmpT, and CroP) in bacterial pathogens are regulated by PhoP/PhoQ. Therefore, we first determined whether PhoP is predicted to bind within the *pla* promoter. The consensus sequence for the PhoP-DNA-binding interaction has been defined in *Salmonella*, *E. coli*, and *Y. pestis* with high homology between them. The consensus sequence determined in *Y. pestis* is two TGTTTAW heptameric repeats separated by four nucleotides (17). We identified a putative PhoP-binding box displaying high homology (15/18 nucleotides) within the core promoter region of *pla* (Fig. 1). The position of this putative-binding box is similar to the PhoP-binding site in the promoter of *Y. pestis rovA,* where a PhoP-binding box overlaps the core promoter and transcription start site (21). While PhoP-binding boxes are generally found further upstream of the transcriptional start site of target genes, this binding box position is also documented in multiple *Salmonella* genes thought to be repressed by PhoP upon PhoP/PhoQ activation (23). The binding box for Crp, a transcriptional activator of *pla*, is upstream of the putative PhoP-binding box (Fig. 1). Crp-mediated induction of *pla* expression occurs during the later stages of pneumonic plague in response to decreasing glucose concentrations in the lungs (24), and the location of the putative PhoP-binding box suggests that PhoP may interfere with Crp activation of *pla*. Based on the location of the predicted binding box and similarity to PhoPQ-repressed genes, we predict that *pla* expression is negatively regulated by PhoP/PhoQ.

### PhoP binds a putative PhoP-binding box within the core *pla* promoter

**Fig 2:**
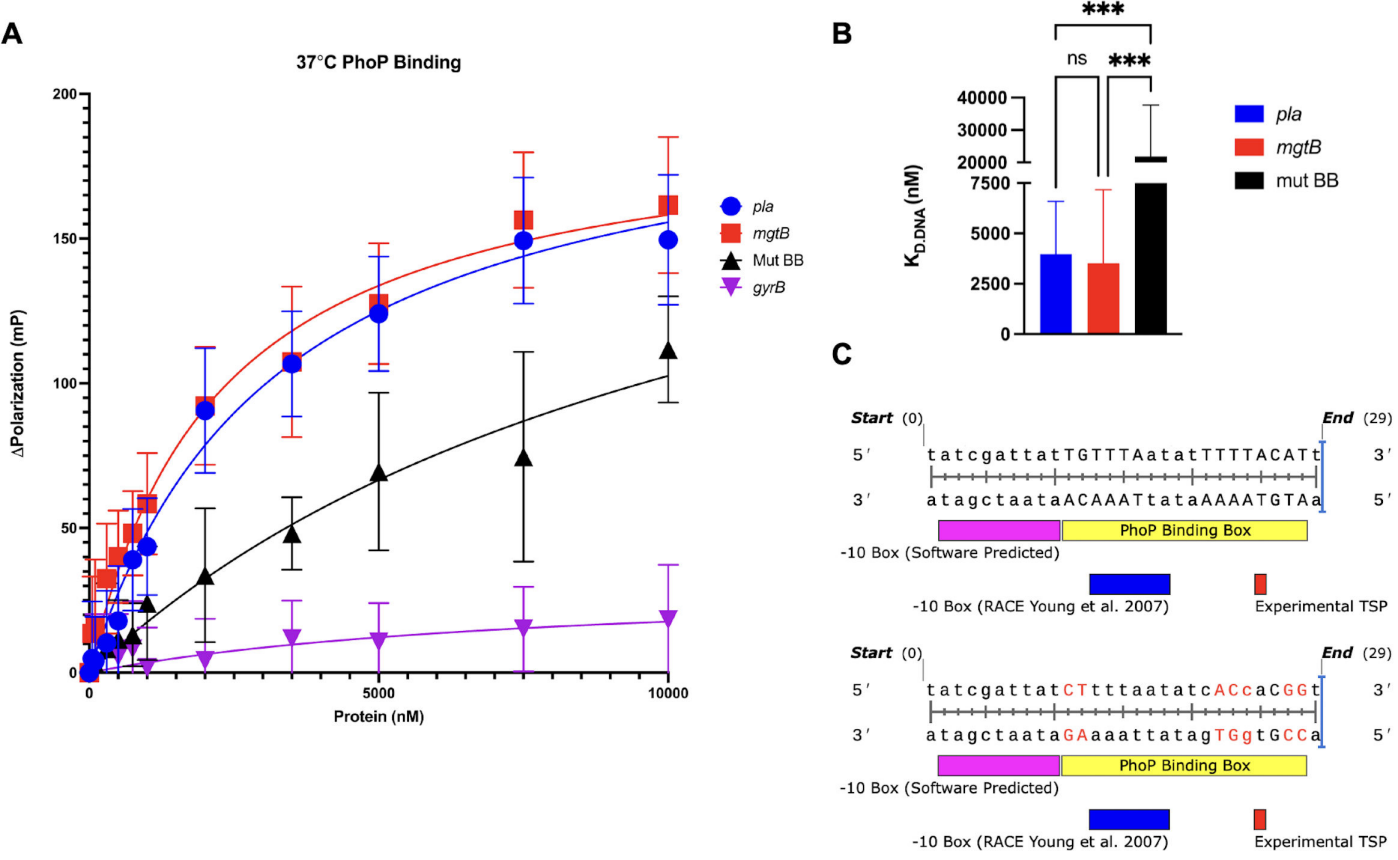
PhoP specifically binds a putative PhoP-binding box within the core *pla* promoter. Fluorescence anisotropy analysis of recombinant PhoP binding to PhoP-binding box sequence. (**A**) rPhoP was titrated into a solution containing FAM-labeled positive control ds-*mgtB* (red), ds-*pla* (blue), ds-mut BB (black), or negative control ds-*gyrB* (purple) DNA oligonucleotides (1 nM) and 1% vol/vol PEG8K at 37°C. (**B**) Fluorescence polarization changes were measured and fit to a quadratic equation to yield displayed equilibrium dissociation constants. Values reported for ds-*pla*, ds-*mgtB*, and ds-mut BB are from three independent experiments. (**C**) Schematic of changes made for ds-mut BB sequence within the pla promoter. The putative PhoP-binding box is designated in all caps for A and B; mutations made to the binding box are in red font in the bottom panel. ns, not significant; ***, *P* ≤ 0.001. *P*-values were calculated using a one-way ANOVA.

We next sought to determine whether the predicted binding box within the *pla* promoter can be bound by PhoP using fluorescence anisotropy. To evaluate the binding capacity of PhoP to the putative PhoP-binding box, changes in fluorescence polarization were monitored for fluorescein amidite (FAM)-labeled ds-DNA substrates when incubated with recombinant PhoP (rPhoP) as a function of protein concentration, and the resulting data were fit to a quadratic equation to obtain estimated equilibrium dissociation constants (K_D.DNA_). 38 nt 5′-FAM-labeled oligonucleotides containing the consensus PhoP box sequence within the promoter of the known PhoP-regulated gene *mgtB* (positive control) encoding a Mg^+2^ importer, the putative PhoP box sequence within the *pla* promoter, or the promoter sequence of *gyrB* (negative control) were incubated with increasing concentrations of unphosphorylated *Y. pestis* rPhoP and subjected to polarized light. Binding was assayed at 37°C at a DNA concentration of 1 nM with the use of PEG8K (polyethylene glycol) as a crowding agent to promote DNA-protein interaction. Evaluation of rPhoP binding capacity to the ds-DNA substrates revealed clear and near-identical affinity for both ds-*pla* and ds-*mgtB,* with no significant difference in K_d_ (Fig. 2A and B), indicating that PhoP binds the putative-binding box sequence upstream of *pla* similar to the sequence of a known PhoP-regulated gene. In contrast, rPhoP displayed little, if any, affinity to the negative control ds-*gyrB* (Fig. 2A and B). Due to the complete lack of binding affinity between rPhoP and ds-*gyrB*, accurate K_D_ values could not be calculated and are therefore not shown. We also generated a labeled putative PhoP box sequence from within the pla promoter with non-essential nucleotides altered in both heptameric repeats (ds-mut BB) to determine whether mutating the *pla*-binding box altered PhoP binding (Fig. 2C). Altering the putative-binding box sequence resulted in significantly decreased binding of rPhoP as evidenced by an increased K_d_ (Fig. 2A and B). These results indicate that PhoP binds the putative-binding box sequence found within the *pla* promoter, and this binding was dependent on the predicted PhoP-binding box.

### *pla* is regulated by PhoP under PhoP-/PhoQ-inducing conditions in *Y. pestis in vitro*

**Fig 3:**
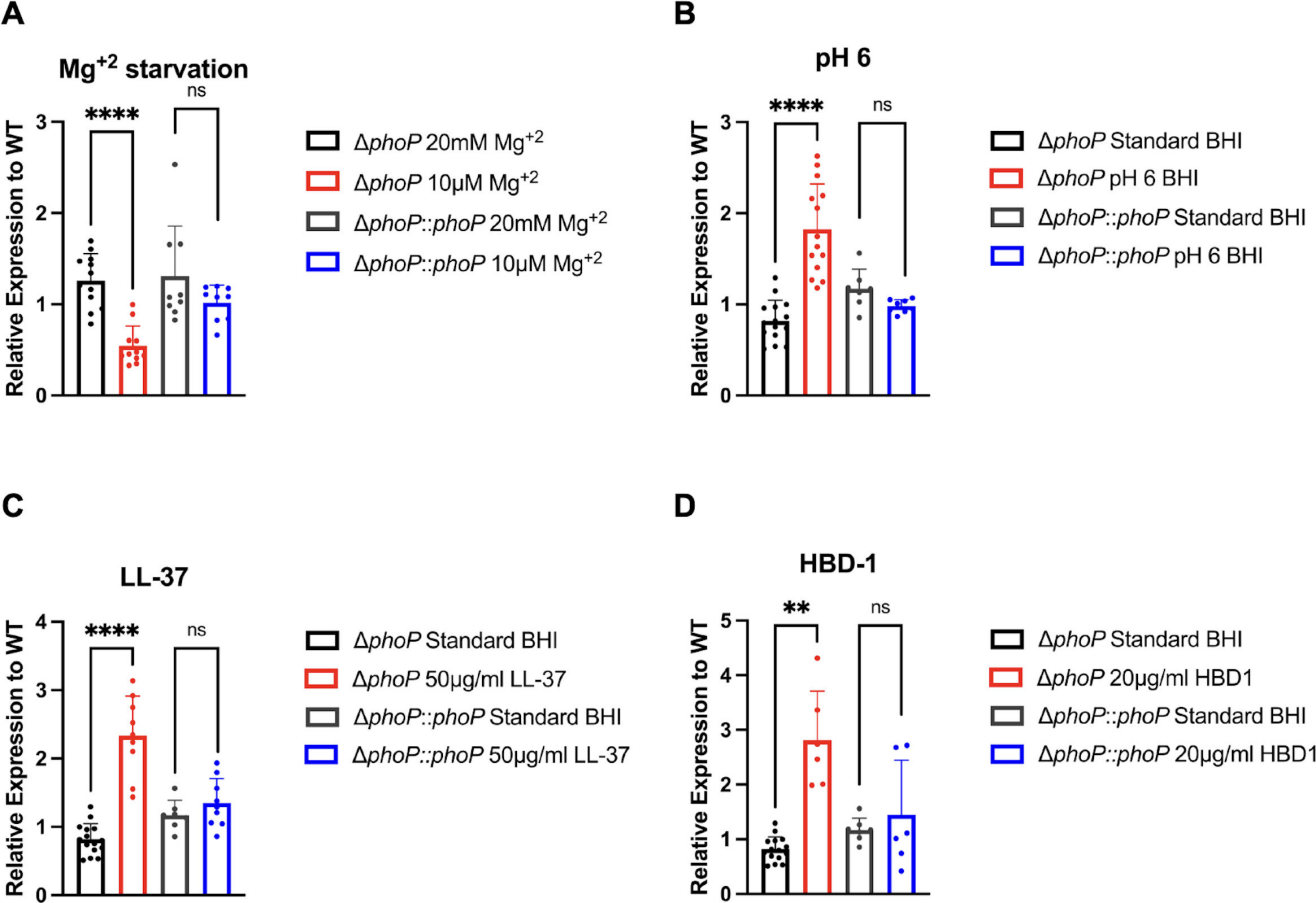
PhoP regulates *pla* expression under Pho-P/PhoQ-inducing conditions. Panels display relative expression of *pla* in Δ*phoP* or Δ*phoP* complemented with WT *phoP* (D*phoP::phoP*) vs WT bacteria (**A**) in PMH minimal media containing 20 mM or 10 μM Mg^+2^ for 2 h at 37°C, or in complex BHI media with (**B**) pH 6 BHI, (**C**) 50 μg/mL LL-37, or (**D**) 20 μg/mL HBD1 for 2 h at 37°C. Fold change was calculated using the ΔΔCT method to compare the expression of *pla* in samples grown under each PhoP-/PhoQ-inducing condition compared to “uninduced” standard culture conditions. Each sample was normalized to *gyrB*, and fold change is plotted as *pla* expression in mutant or complemented strain compared to WT bacteria for each culture condition. ns, not significant; **, *P* ≤ 0.01; ****, *P* ≤ 0.0001 (Welch’s *t*-test). *N* = 15 (pH 6), *N* = 12 (10 μM Mg^+2^), *N* = 9 (LL-37), and *N* = 6 (HBD-1). Baseline expression of *pla* in Δ*phoP* vs WT bacteria at 37°C in BHI is displayed as standard BHI or 20 mM Mg^+2^.

The PhoP/PhoQ TCS aids bacteria in adapting to hostile environments by coordinately altering gene expression in response to varying environmental stimuli (25). PhoP-/PhoQ-inducing stimuli in *Y. pestis* and related gram-negative pathogens such as *Salmonella* and *E. coli* include low Mg^+2^ (<10 µM), low pH, and CAMPs, and PhoP-/PhoQ-mediated regulation of omptin proteases has been reported under similar conditions (10). Therefore, we sought to evaluate whether PhoP/PhoQ alters *pla* expression under PhoP-/PhoQ-inducing conditions. We generated a strain of *pgm^–^ Y. pestis* Colorado 92 (CO92) lacking *phoP* (Δ*phoP*), as well as the corresponding mutant strain (Δ*phoP*) complemented with the PhoP open reading frame (ORF) and associated native promoter (Δ*phoP::phoP*), and evaluated *pla* expression in each strain. PhoP/PhoQ is known to regulate numerous genes in Mg^+2^-depleted environments (10, 14, 17, 18, 23). To examine *pla* expression, wild type (WT), Δ*phoP,* and Δ*phoP::phoP* strains grown in magnesium-replete (20 mM Mg^+2^) Piperazine-N,N′bis(2-ethanesulfonic acid)-HEPES-buffered minimal medium (PMH) were diluted to equal optical densities and grown for 2 h at 37°C in PMH media containing 10 μM Mg^+2^ (low magnesium). Following treatment, samples were analyzed by quantitative reverse transcription (qRT)-PCR, and *pla* expression was compared between magnesium-replete and -deplete conditions. When grown in media containing 10 μM Mg^+2^, *pla* expression was significantly reduced in the Δ*phoP* mutant relative to WT when compared to growth in PMH containing 20 mM Mg^+2^, indicating that PhoP induces *pla* expression under this condition (Fig. 3A). These results are consistent with known examples of PhoP/PhoQ regulation of omptin proteases (10–12). Next, we evaluated *pla* expression by bacteria grown at low pH (6) as a PhoP/PhoQ inducer for 2 h at 37°C. In contrast to growth under low Mg^+2^, we observed a significant increase in *pla* expression in the Δ*phoP* mutant relative to WT bacteria when compared to growth in standard brain-heart infusion (BHI) broth (Fig. 3B), suggesting that PhoP suppresses *pla* expression under this condition. This aligns more closely to what is expected with the placement of the PhoP-binding box and its overlap with the −10 region and transcriptional start site of *pla*. Finally, we tested whether the presence of CAMPs alters *pla* expression. When treated with 50 μg/mL LL-37, which is found in both macrophages and neutrophils, and 20 μg/mL human beta defensin-1 (HβD-1), which is produced by respiratory tract epithelial cells, we observed a significant increase in *pla* expression in the Δ*phoP* mutant relative to WT bacteria when compared to growth in standard BHI broth (Fig. 3C and D), again suggesting that PhoP suppresses *pla* expression under these conditions. When Δ*phoP* was complemented with the WT *phoP* gene, no significant difference was observed relative to WT under any conditions. These results indicate that *pla* is repressed in WT bacteria upon PhoP/PhoQ activation by low pH and CAMPs, while *pla* expression is elevated in a Mg^+2^-deplete environment.

### Mutating the PhoP-binding box eliminates *pla* regulation by PhoP in the presence of low pH *in vitro*

**Fig 4:**
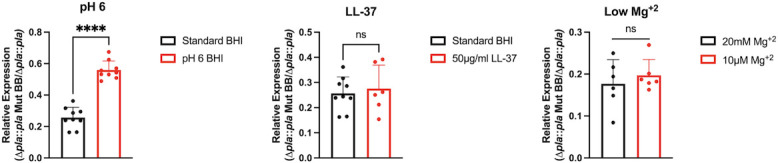
PhoP binding to the *pla* promoter represses *pla* in *Y. pestis* in the presence of low pH *in vitro*. Relative expression of *pla* in the Δ*pla*::D*pla* PhoP Mut BB strain relative to Δ*pla*::D*pla* after growth in standard BHI, pH 6 BHI, BHI containing 50 μg/mL LL-37, or PMH minimal media containing 20 mM or 10 μM Mg^+2^ for 2 h at 37°C. Fold change was calculated using the ΔΔCT method. ns, not significant; ****, *P* ≤ 0.0001 (Welch’s *t*-test). *N* = 9 (pH 6), *N* = 6 (LL-37 and 10 μM Mg^+2^).

While the fluorescence anisotropy data suggest that PhoP can directly bind the putative-binding box within the *pla* promoter, it remains possible that the regulation of *pla* expression by PhoP is indirect through its impact on other transcriptional regulators. To determine whether PhoP directly binds to the putative PhoP-binding box to alter *pla* expression, we complemented the Δ*pla* strain with the *pla* gene/promoter containing a mutated PhoP-binding box (Mut BB) identical to the oligonucleotide sequence used in Fig. 2. The Mut BB strain was directly compared to a single-copy Δ*pla::pla* strain *in vitro* in the presence of low pH, LL-37, and low Mg^+2^. We predicted that if direct PhoP binding alters *pla* expression, mutating the PhoP-binding box would impact the expression of *pla* similar to the deletion of PhoP (Δ*phoP*). After the growth of both strains for 2 h at 37°C in pH 6 BHI and qRT-PCR analysis, we observed a ~2-fold increase in *pla* expression in the PhoP Mut BB strain relative to the Δ*pla::pla* strain (Fig. 4). This is strikingly similar to what was observed with the Δ*phoP* strain under identical conditions (Fig. 3B). This result, combined with those of Fig. 3, suggests PhoP directly binds our putative-binding box and alters *pla* expression in response to low pH. Induction of PhoP/PhoQ with LL-37 and low Mg^+2^ stress, though, did not impact *pla* expression in the Mut BB strain compared to Δ*pla::pla* (Fig. 4). Of note, under baseline conditions in media alone, the Mut BB strain exhibited reduced *pla* expression relative to the Δ*pla::pla* strain, indicating that mutation of the promoter region that includes the PhoP-binding box altered *pla* expression in uninduced conditions and likely complicated the analysis. In summary, mutating the putative PhoP-binding box alleviated the repression of *pla* under low pH conditions but did not impact the other conditions tested. Mutating the PhoP-binding box also resulted in decreased *pla* expression in standard complex media, further highlighting the importance of this particular sequence.

### Pla protein levels are reduced upon PhoP/PhoQ activation by low pH and CAMPs

**Fig 5:**
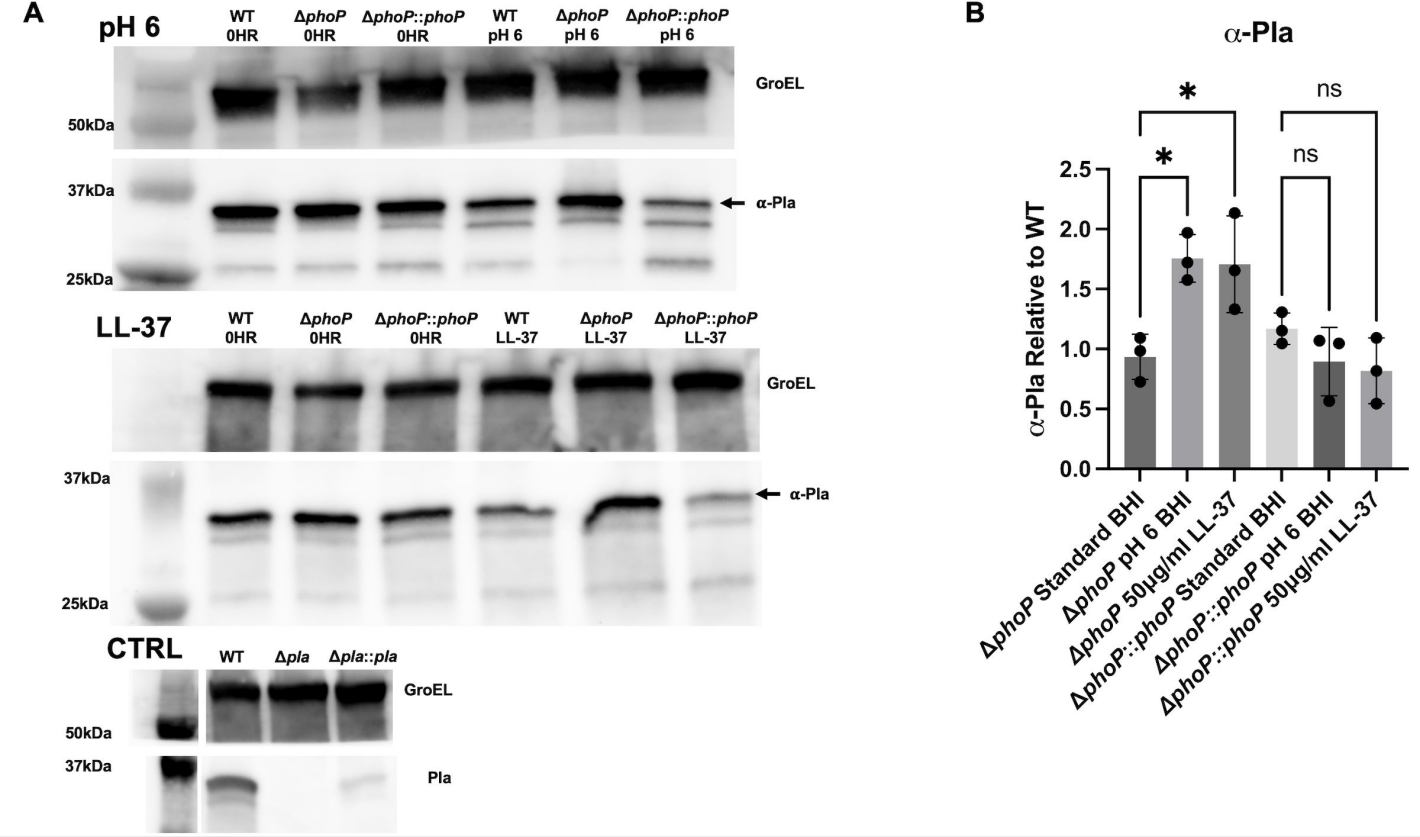
PhoP reduces Pla protein expression in WT *Y. pestis*. (**A**) Representative blots of protein lysates from pH 6 and 50 μg/mL LL-37-treated cultures probed with anti-Pla rat serum and anti-GroEL antibody. Uninduced/untreated samples for each strain are in the first three lanes of each blot, and treated samples are in the last three. The loading control GroEL is shown above Pla for both pH 6 and LL-37. Lane 1—Ladder, Lane 2—WT uninduced, Lane 3—Δ*phoP* uninduced, Lane 4—Δ*phoP::phoP* uninduced, Lane 5—WT pH 6/LL-37, Lane 6—Δ*phoP* pH 6/LL-37, and Lane 7—Δ*phoP::phoP* pH 6/LL-37. Control blot probing lysate from Δ*pla* and complemented mutant for both GroEL and Pla is also shown. (**B**) Densitometry analysis of average α-Pla (35 kDa)/GroEL ratio from three different Western Blots of protein lysates from Δ*phoP* or Δ*phoP::phoP* strains relative to WT in standard BHI, pH 6 BHI, or 50 μg/mL LL-37. Ratios ns, not significant; *, *P* ≤ 0.05 (one-way ANOVA). *N* = 3.

We next sought to validate the findings that *pla* expression is reduced upon PhoP/PhoQ activation at the protein level. WT, Δ*phoP*, and Δ*phoP::phoP* bacteria were cultured identically to the qRT-PCR analysis, and cell lysates were collected for Western blot analysis using rat sera generated against full-length Pla. Respective images of blots from uninduced, pH 6 BHI, and LL-37 samples were analyzed using densitometry to quantify Pla levels (Fig. 5A). On measuring α-Pla using densitometry, the predominant form of Pla found in the outer membrane, we observed a significant increase in Pla production in the Δ*phoP* mutant relative to WT under low pH and LL-37-inducing conditions when compared to protein concentrations of uninduced cultures (Fig. 5B). Analysis of total Pla, which includes autoproteolytic derivatives, β-Pla (33 kDa) and γ-Pla (31 kDa) in addition to α-Pla (35 kDa), showed near identical results (Fig. S1). This change was not apparent in the Δ*phoP::phoP* strain for either low pH or LL-37-treated cultures, confirming that the loss of PhoP was responsible for the change. We also evaluated Pla expression in the presence of 10 μM Mg^+2^ and 20 μg/mL HβD-1, in addition to a Δ*pla* strain to control for non-specific banding. Treatment with 20 μg/mL HβD-1 resulted in a similar increase in detected Pla, while 10 μM Mg^+2^ resulted in no observed change in Pla when compared to cultures grown in 20 mM Mg^+2^ (data not shown). These results, in line with data from Fig. 3 and 4, suggest that PhoP represses *pla* at the transcriptional level and subsequently reduces protein levels via PhoP/PhoQ sensing of low pH and CAMPs.

### PhoP interferes with Crp-mediated *pla* expression

**Fig 6:**
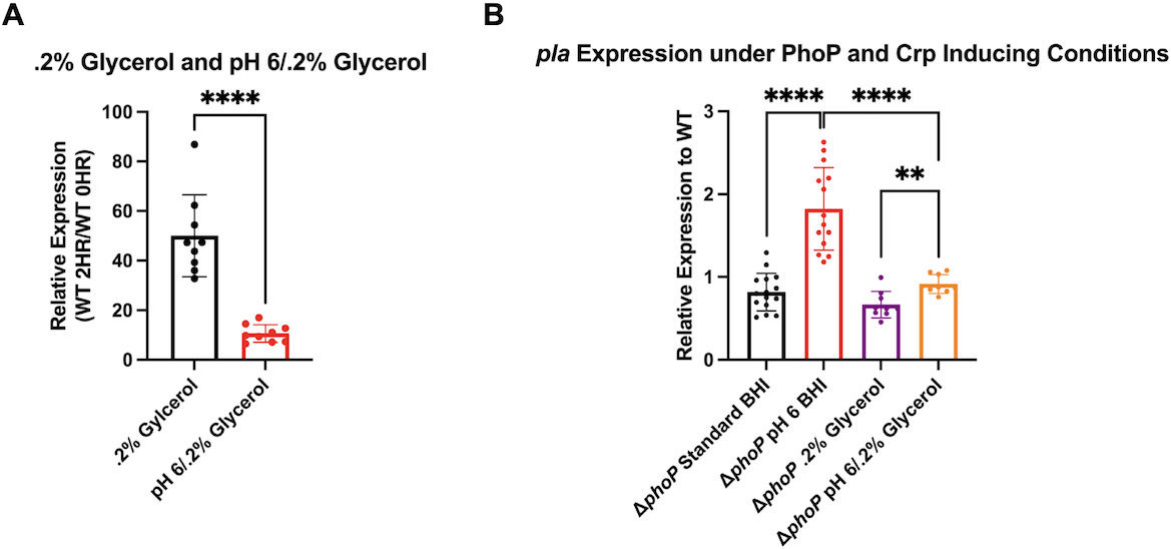
PhoP interferes with Crp-mediated *pla* expression in WT *Y. pestis*. (**A**) Panel displays relative expression of *pla* in WT bacteria grown for 2 h at 37°C in PMH minimal media containing 0.2% glycerol or pH 6 PMH minimal media containing 0.2% glycerol vs WT bacteria grown in complex BHI. (**B**) Panel displays relative expression of *pla* in Δ*phoP* or Δ*phoP* complemented with WT *hoP* (D*phoP::phoP*) vs WT bacteria in complex BHI media, pH 6 BHI, PMH minimal media containing 0.2% glycerol, or pH 6 PMH complete minimal media containing 0.2% glycerol for 2 h at 37°C. Fold change was calculated using the ΔΔCT method. **, *P* ≤ 0.01; ****, *P* ≤ 0.0001 (Welch’s *t*-test). *N* = 9 (0.2% glycerol and pH 6/0.2% glycerol). Baseline expression of *pla* in Δ*phoP* vs WT bacteria at 37°C in BHI is displayed as standard BHI.

Expression of *pla* is positively regulated by Crp under low glucose growth conditions, which occurs in the lungs during the later stages of pneumonic plague (24). We predict that PhoP suppresses *pla* expression by binding the promoter downstream of the Crp-binding box, suggesting that the two regulators may work conversely. We therefore sought to determine how PhoP alters Crp-mediated *pla* expression by inducing both regulators simultaneously. Bacteria were cultured in PMH minimal media containing 0.2% glycerol (to induce Crp) in place of glucose under neutral pH, or in media containing 0.2% glycerol with low pH (pH 6 to induce PhoP) for 2 h at 37°C, and *pla* expression was analyzed by qRT-PCR. As predicted, incubation of WT bacteria in media containing 0.2% glycerol in place of glucose to induce Crp resulted in increased *pla* expression compared to cultures grown in the presence of glucose (Fig. 6A). Growth under both conditions, low pH and 0.2% glycerol simultaneously, resulted in significantly reduced *pla* expression when compared to growth in neutral pH media only lacking glucose (Fig. 6A). We observed significantly increased *pla* expression in the Δ*phoP* mutant relative to WT when grown in pH 6 PMH minimal media containing 0.2% glycerol compared to *pla* expression of the Δ*phoP* mutant relative to WT grown in PMH minimal media containing 0.2% glycerol (Fig. 6B). These results suggest that both PhoP and Crp can act on the *pla* promoter simultaneously, and PhoP binding diminishes Crp-mediated expression of *pla*. Conversely, Crp activation can partially overcome PhoP-mediated suppression of *pla*.

## DISCUSSION

In this study, we show that the production of the essential *Y. pestis* virulence factor Pla is regulated by the PhoP/PhoQ TCS for the first time, which may have implications in our understanding of *pla* regulation during the progression of pneumonic plague. We identified a putative PhoP-binding box within the *pla* core promoter region and verified PhoP binding by fluorescence anisotropy. This is in contrast to other PhoP-regulated omptin-family proteases, such as *pgtE, ompT, ompP,* and *croP,* where PhoP binds upstream of the core promoter region (10, 23). Induction of the PhoP/PhoQ system using low pH or CAMPs resulted in *pla* being repressed in WT bacteria, while, unexpectedly, *pla* transcription was elevated in WT bacteria exposed to Mg^+2^-deplete conditions suggesting that PhoP may differentially regulate *pla* expression depending on the environmental stimulus.

PhoP-/PhoQ-mediated regulation of omptin family proteases is well documented in other gram-negative pathogens, such as *E. coli*, *S. typhimurium*, *S. flexneri*, and *C. rodentium* (10, 11, 26, 27). Known PhoP-/PhoQ-regulated genes expressing omptin proteases typically contain a PhoP-binding box upstream of the core promoter that, when bound, results in increased expression. We identified a PhoP-binding box that resides within the core promoter of *pla* overlapping with the −10 site, differing from the typical box placement for regulation of omptin proteases in many related gram-negative pathogens. The location of the PhoP-binding box, within the core promoter region, implies that *pla* may be repressed upon PhoP binding. This is the case with other PhoP-regulated genes, such as *rovA* in *Y. pestis* and *pagK* and *ugtL* in *S. typhimurium,* that contain PhoP-binding boxes within the core promoter region (21, 23). Additionally, the location of the PhoP-binding box is ~50 nts downstream of the Crp-binding box. Crp-mediated *pla* expression is significantly elevated during the pro-inflammatory phase of pneumonic plague, where it is predicted that the reduced available glucose within bacterial microcolonies activates Crp (24). This close proximity suggests that the PhoP and Crp systems may interact and/or counteract each other to finely tune the regulation of *pla*.

Utilizing fluorescence anisotropy, we show that the native PhoP-binding box sequence located within the *pla* promoter can be bound by *Y. pestis* PhoP with specificity and does so with an affinity comparable to the full consensus sequence PhoP-binding box found in the promoter of the PhoP-regulated gene *mgtB*. The *pla* PhoP-binding box contains no mismatches within the first heptameric repeat, but contains three mismatches within the second heptameric repeat when compared to the *Y. pestis* PhoP-binding consensus sequence TGTTTAW-N_4_-TGTTTAW (17). This binding box composition aligns with previously reported interaction bias within the first heptameric repeat, and ablation of the repeats by altering nucleotides not part of the −10 box or TSS significantly reduced the binding capacity of rPhoP to the sequence.

PhoP-/PhoQ-mediated regulation of omptin proteases has been verified across multiple species, but not for *Y. pestis*. The PhoP/PhoQ system responds to environmental stimuli such as changes in Mg^+2^ concentration, pH, and the presence of CAMPs (14–16, 28), and the proteases *ompP* and *ompT* of *E. coli, pgtE* of *S. typhimurium,* and *croP* of *C. rodentium* display positive regulation by PhoP under varying environmental stimuli (10–12). When treated with low pH or CAMPs *pla* expression is reduced in WT *Y. pestis* when compared to the Δ*phoP* strain. This differs from related omptin proteases regulated by PhoP/PhoQ but agrees with the position of the PhoP-binding box described in Fig. 1. While the relative fold changes across the conditions tested may be considered rather modest in the twofold to fourfold range, this is not necessarily surprising for *Y. pestis* virulence determinants. Previous work showed that the expression of even essential *Y. pestis* virulence factors such as components of the Ysc T3SS and the *Yersinia* outer proteins (Yops), was upregulated twofold to sixfold during both *in vitro* and *in vivo* infection compared to laboratory-grown culture (29, 30). Unexpectedly, when treated with 10 μM Mg^+2^ and compared to media containing 20 mM Mg^+2^, *pla* expression is elevated in WT *Y. pestis* when compared to the Δ*phoP* strain. While *pla* is positively regulated by PhoP under low Mg^+2^ conditions much like other omptin proteases, this contrasts with the regulation in response to low pH and CAMPs. It is unclear how sensing low Mg^+2^ stress by PhoP/PhoQ would impact *pla* expression differently than other stimuli. PMH minimal medium used to modulate Mg^+2^ concentration has a reduced glucose concentration compared to complex BHI medium. As Crp expression is also modulated by PhoP, the reduced glucose potentially results in elevated intracellular cAMP levels that activate CRP and increase *pla* expression, essentially outcompeting PhoP-mediated repression. Additionally, another yet-to-be-identified regulator may be modifying PhoP-P levels or altering *pla* expression either directly or indirectly. It is conceivable that under magnesium-deplete conditions and not the other conditions tested, a specific regulator or regulatory network is simultaneously induced that converges with the PhoP/PhoQ system to differentially regulate *pla*. While this finding needs to be verified, the potential for differential directional regulation of target genes by PhoP that depends on the stimulus is intriguing and may highlight an understudied feature of the PhoP/PhoQ and other TCSs.

Generation of a Δ*pla::pla* PhoP Mut BB strain permitted specific and targeted evaluation of the transcriptional response to PhoP-mediated repression. While the phenotype observed in response to pH stress matched that of the Δ*phoP* mutant, the same was not observed for both low Mg^+2^ and CAMP-induced stress. It is currently difficult to conclude anything from this, as mutating the PhoP- binding box resulted in reduced expression of *pla* in culture. Furthermore, complementation with the *pla* PhoP Mut BB locus occurs in a single copy via integration in a neutral site within the chromosome, as opposed to its native location on pPCP1, potentially impacting copy number. Complementing *pla* in single copy, though, restores the virulence of mutant strains comparable to WT bacteria, and therefore, it is not clear whether this might impact our analysis (8). Still, a reduction in DNA accessibility, due to the non-native chromosomal integration of the *pla* PhoP Mut BB, or alteration of the PhoP- binding box within the *pla* promoter potentially impacted transcription, although the −10 box, TSS and other promoter elements remained unchanged. Interestingly, the phenotype disparity observed between low pH, low Mg^+2^, and CAMP-treated samples reflects the spatially separate PhoQ stimuli recognizing domains (28, 31, 32). PhoQ responds to changes in cations and the presence of CAMPs via the periplasmic domain, while the cytoplasmic domain responds to a decrease in cytoplasmic pH (28, 31, 32). Whether stimulus-specific activation of PhoQ impacts PhoP-P activity, abundance, or both remains unknown. Regardless, while our data suggest direct regulation of pla by PhoP, we cannot rule out the possibility that PhoP/PhoQ impacts the expression of other regulators of *pla* that either work in concert with PhoP or independently to regulate *pla* expression.

Analysis of Pla protein concentration by Western blotting revealed a significant increase of α-Pla in the Δ*phoP* mutant relative to WT under low pH and CAMP stress, in line with our reported qRT-PCR results. Interestingly, growth under low Mg^+2^ stress did not increase Pla concentration in WT bacteria. This may, though, be due to limitations in the resolution/sensitivity of quantifying Western blot analysis, and a proteomics approach may be necessary to more accurately evaluate the impact of loss of PhoP on Pla production. Three isoforms of Pla have been identified—α-Pla (35 kDa), β-Pla (33 kDa), and γ-Pla (31 kDa) (33, 34). γ-Pla was not detected as this isoform is an alternate processing of α-Pla and disappears when cell lysates are boiled in the presence of sodium dodecyl sulfate (SDS) (34). α-Pla and β-Pla do not differ in enzymatic activity, and both are integral outer membrane proteins (35). The integral nature of Pla, combined with the necessity for bacterial outgrowth in the lung compartment, highlights a potentially complex regulation scheme employed by *Y. pestis* for maximal enzymatic activity while limiting immune detection, likely during the pre-inflammatory phase of infection.

**Fig 7:**
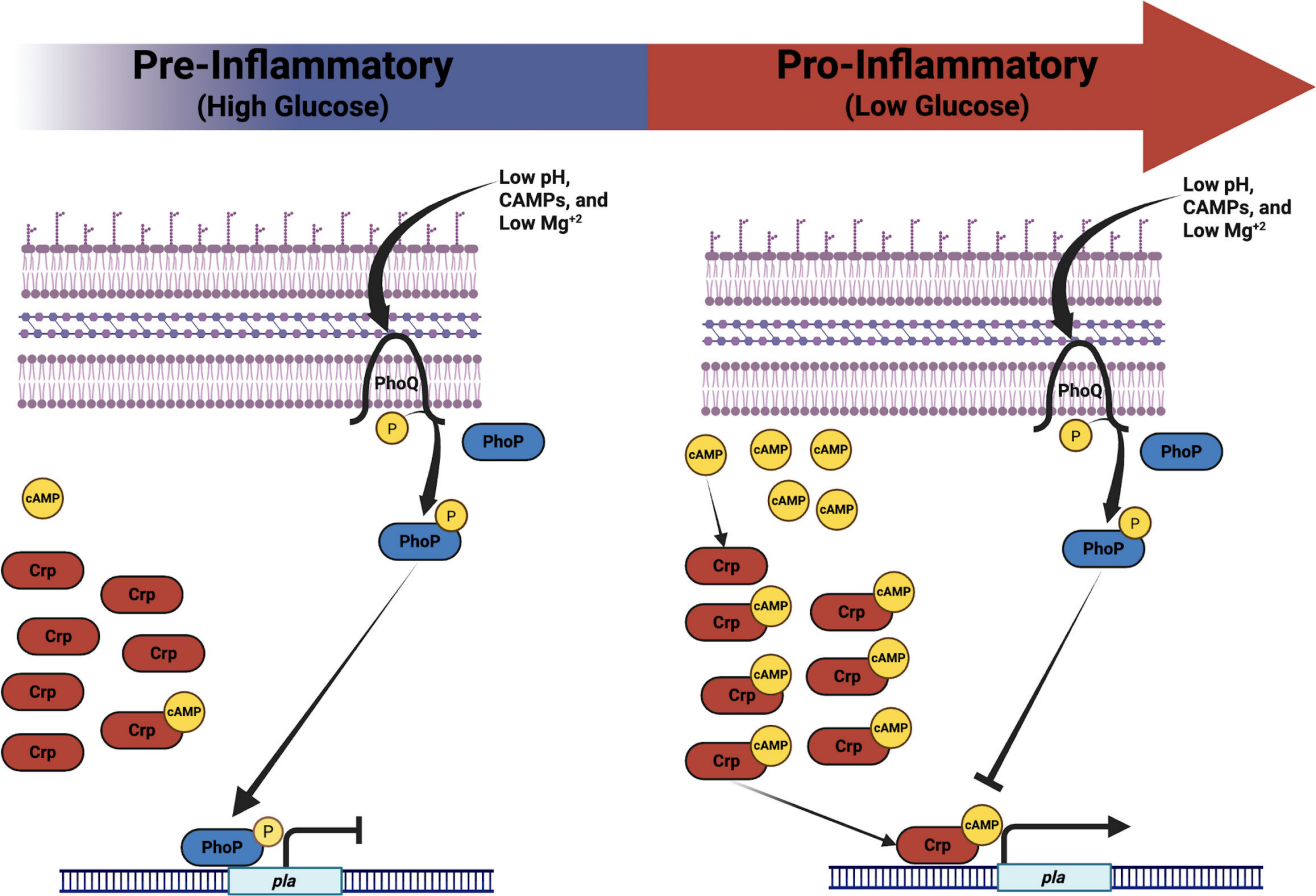
The *Y. pestis* plasminogen activator protease is regulated by the PhoP/PhoQ two-component system. (Left) During the pre-inflammatory phase of pneumonic plague, *Y. pestis* encounters an environment containing sufficient glucose concentrations to prevent activation of Crp (red) and subsequent upregulation of *pla*, while encountering PhoP/PhoQ-inducing stimuli that activate PhoP (blue) and lead to repression of *pla*. (Right) Following the shift to the pro-inflammatory phase of pneumonic plague and a rapid influx of immune cells into the pulmonary compartment, glucose concentrations drop, prompting an increase in intracellular cAMP, activation of Crp (Crp-cAMP), and upregulation of *pla* by overriding PhoP-mediated repression.

Previous work showed that the expression of *pla* is dramatically reduced during the early stages (<48 hpi) compared to the later stages of infection (36). It is clear, though, from our work and others that Pla is essential to bacterial survival in the lungs during this stage (7, 8). During the later stages of pneumonic plague, Crp-mediated *pla* expression is driven by an increasingly glucose-deplete pulmonary environment that leads to elevated intracellular cAMP concentrations and Crp-cAMP-driven *pla* transcription (22, 24, 36, 37). In the context of disease progression, this corresponds with the shift from the pre-inflammatory to the pro-inflammatory phase of infection as immune cell infiltration into the pulmonary compartment prompts a substantial decrease in available glucose (24). Aside from Crp, the factors responsible for the dynamic regulation of *pla* during the biphasic progression of pneumonic plague are not understood. We propose a model where early during infection, PhoP is activated to limit the expression of *pla* and prevent promiscuous and potentially immunogenic cleavage of Pla substrates (38). To evaluate the impact of PhoP on pla expression, we incubated bacteria under conditions predicted to be encountered during mammalian infection at 37°C. At steady state, the pH of the human upper respiratory tract is acidic (5.5–6.5), while the lower respiratory tract is slightly less acidic, being a pH of 6.5–7 (39, 40). The production of CAMPs, such as HβD-1, by respiratory epithelial cells in the nasal passages, trachea, and bronchi also occurs at steady state (41). We posit that signals such as these act as environmental stimuli that *Y. pestis* may encounter during the pre-inflammatory phase of pneumonic plague. Additional signals, such as LL-37, produced by neutrophils and macrophages, and other CAMPs, produced by resident or recruited cells, may also contribute to stimuli that regulate *pla* during the pre-inflammatory phase (41, 42). As the disease progresses into the pro-inflammatory phase and upon sensing a marked reduction in available glucose, PhoP-mediated repression is overcome by increasing levels of Crp-cAMP, thus driving *pla* transcription (Fig. 7). Expression of Crp is also induced by PhoP, potentially further priming the Crp system during the pre-inflammatory phase of disease in advance of its activation in the presence of low glucose (43, 44). Of note, a potential limitation to the current study is the focus on the regulation of *pla* at mammalian body temperature in calcium-enriched media and under conditions we anticipate are encountered during pulmonary infection. It is yet to be determined whether PhoP regulates *pla* at low-calcium concentrations or at 26°C under conditions that are found in the flea vector, in particular within the biofilm that facilitates flea-borne transmission of bacteria resulting in bubonic plague.

In summary, we show that the expression of the essential *Y. pestis* virulence factor Pla is regulated by the PhoP/PhoQ two-component system via a putative PhoP-binding box located within the −10 box and transcription start site. On its surface, the reduction of the expression of a critical virulence factor, such as *pla*, in response to host-derived stimuli appears counterintuitive. Given the importance of Pla to *Y. pestis* virulence, its activation by PhoP/PhoQ, which is activated under conditions typically found *in vivo*, would have been a more likely prediction. In the context of pneumonic plague, *pla* is essential for early bacterial growth in the lungs and is responsible for limiting neutrophil infiltration into the lungs and resistance to neutrophil-mediated killing (7, 8). Due to their pleiotropic effects, surface proteases must be tightly regulated. This has been demonstrated recently in *Staphylococcus aureus*, where, though extracellular proteases are important virulence factors, limiting their expression is equally important to prevent promiscuous cleavage of other virulence factors or host determinants that can lead to innate immune response and increased attenuation (45). We posit that Pla, as a promiscuous outer membrane protease and essential virulence factor, requires multiple modes of regulation for finely tuned expression and maximized effectiveness while limiting host detection and subsequent inflammatory responses that could result from aberrant cleavage of bacterial and host substrates to maintain a pre-inflammatory phase during pneumonic plague.

## MATERIALS AND METHODS

Bacterial strains, *pgm^–^* CO92 *Y. pestis* and *pgm^–^* D*phoP* CO92 *Y. pestis,* were grown on BHI agar (Difco Laboratories) at 26°C for 2 days. For PhoP/PhoQ induction experiments, liquid cultures of WT and Δ*phoP* CO92 *Y. pestis* were grown overnight in 10 mL BHI broth containing 2.5 mM calcium chloride at 37°C with constant shaking. Mutant bacterial strains were generated in the *pgm^–^* CO92 *Y. pestis* background, which is referred to as “wild type” throughout the study. The Δ*phoP pgm^–^* CO92 was generated by lambda red recombination as described previously (46). Briefly, 500 bp fragments directly flanking *phoP* and the kanamycin resistance (Kan^R^) cassette from pKD13 were amplified by PCR, and these fragments were fused by splicing overlap extension PCR (SOE-PCR) to generate a 2.4 kb allelic exchange product. The allelic exchange product was electroporated into fully virulent CO92 *Y. pestis* carrying pWL204 (8), which contains the lambda red recombination machinery, and recombinant clones were selected on Kan 50 μg/mL (Kan^50^) plates and confirmed by PCR. pWL204 was cured from recombinant clones by culturing on BHI plates containing 5% sucrose. The plasmid pSKIPPY, which contains the FLP recombinase gene, was electroporated into recombinant clones, and clones were subsequently cultured overnight at 26°C in the presence of 1 mM isopropyl β-d-1-thiogalactopyranoside (IPTG) to resolve the kanamycin cassette and leave a 27aa scar sequence in frame with the surrounding genetic loci. Following resolution, loss of *phoP* was confirmed by the presence of a 1 kb product.

Complementation of *phoP* and *pla* and the Δ*pla::pla* PhoP Mut BB strain was generated by Tn7 chromosomal integration as previously described (8). For *phoP* complementation, the coding region and upstream intergenic region of *phoP* were amplified using primers containing RE sites PstI and BamHI. The *phoP* construct was cloned into the TOPO 2.1 vector according to the TOPO TA Cloning Kit protocol (Invitrogen). The *phoP* construct was subsequently cloned into the pUC18R6K-mini-Tn7-km MCS via PstI and BamHI RE sites. Both pUC18R6K-mini-Tn7-km-*phoP* and pTNS2 were electroporated into *pgm*^–^Δ*phoP* CO92 *Y. pestis,* and transconjugants were selected on Kan^50^ BHI plates. The plasmid pSKIPPY, which contains the FLP recombinase gene, was electroporated into recombinant clones, and clones were subsequently cultured overnight at 26°C in the presence of 1 mM IPTG to resolve the kanamycin cassette. For *pla* complementation, the coding region and flanking intergenic regions were amplified by PCR using primers containing complementary overhangs to the pUC18R6K-mini-Tn7-km MCS. pUC18R6K-mini-Tn7-km was linearized by two rounds of PCR amplification with NEB Q5 high-fidelity polymerase. Linearized pUC18R6K-mini-Tn7-km and amplified *pla* were transformed into chemically competent S17-λpir *E. coli* to generate a circularized pUC18R6K-mini-Tn7-km-*pla* construct. Downstream steps were identical to *phoP* complementation. For *pla* Mut BB, linearization and mutation insertion of the pUC18R6K-miniTn7-km-*pla* construct was achieved using partially overlapping primers containing the desired mutations (adapted from reference [47]). Linearized and mutagenized pUC18R6K-mini-Tn7-kn-*pla* was amplified a second time using the same primers and transformed into chemically competent S17-λpir *E. coli* to generate a circularized pUC18R6K-mini-Tn7-kn-*pla* Mut BB construct. Downstream steps were identical to *phoP* and *pla* complementation.

### Identification of PhoP-binding box and related promoter elements

The full sequence of the CO92 *Y. pestis* plasmid pPCP1 (Accession Number NC_003132) was downloaded from NCBI and imported into SnapGene. The PhoP consensus binding sequence for *Y. pestis* biovar *Microtus* (TGTTTAW-N_4_-TGTTTAW) (17) was input into SnapGene to search for similar DNA sequences, allowing for a mismatch every six bases. Search results returned seven matches on pPCP1, with one match residing within the *pla* promoter. In a similar manner, the CRP (cAMP receptor protein) consensus binding sequence for *Escherichia coli* (TGTGA-N_6_-TCACA) was input into SnapGene, searching for perfect matches (48). Search results returned one match as previously described. The −35, −10, and transcription start site sequences previously described were also annotated (22).

### Expression and purification of recombinant PhoP

To produce recombinant PhoP, the *phoP* ORF was cloned into pET21a (Sigma), and pET21::PhoP was transformed into BL21(DE3)-pLysS (Monserate Biotechnology Group, San Diego, CA). Cultures were induced for 16 h with 0.1 mM IPTG at 20°C in Luria-Bertani (LB) broth supplemented with 0.5% NaCl (adapted from references [49, 50]), and expressed recombinant PhoP includes a C-terminal His_6_ tag. Recombinant protein was affinity-purified with HisPur Ni-nitrilotriacetic acid (NTA) resin (Thermo Scientific, Rockford, IL) under native conditions. Cells were lysed with 2% NP-40 Surfact-Amps detergent solution (Thermo Scientific), 50 mM Tris-HCl (pH 8.0), 600 mM NaCl, and 10% glycerol along with Lysonase and Benzonase (MilliporeSigma, Burlington, MA) per the manufacturer’s instructions. Soluble and insoluble cellular fractions were separated by centrifugation, and the soluble fraction was incubated with the Ni-NTA resin. Following binding, the resin was washed with 10 volumes of 20 mM Tris-HCl (pH 8.0), 500 mM NaCl, 20 mM imidazole, and 10% glycerol, followed by a wash with 10 volumes of 20 mM Tris-HCl (pH 8.0), 500 mM NaCl, 40 mM imidazole, and 10% glycerol. The protein was eluted from the resin with 20 mM Tris-HCl (pH 8.0), 500 mM NaCl, 300 mM imidazole, and 10% glycerol, and 5 mM dithiothreitol (DTT) was added to each eluted fraction. Following purification, fractions containing PhoP-His_6_ were combined, concentrated with an Amicon Ultra-4 mL 10 kDa molecular-weight cutoff (MWCO) centrifugal-filtration unit (MilliporeSigma), and resolved on a HiPrep 16/60 Sephacryl S-200 HR gel filtration column using an ÄKTA pure 25 L1 fast-performance liquid chromatography system (Global Life Sciences Solutions USA LLC, Marlborough, MA). The protein was eluted with 50 mM Tris-HCl (pH 8.0), 100 mM KCl, 5 mM MgCl_2_, 10 mM DTT, and 10% glycerol, and samples of the fractions were analyzed by SDS-polyacrylamide gel electrophoresis (SDS-PAGE) to assess yield (49). Fractions containing recombinant PhoP were pooled and concentrated to approximately 3 mg/mL using an Amicon Ultra-15 mL 10 kDa MWCO centrifugal-filtration unit (MilliporeSigma).

### Generation of Pla recombinant protein and Pla-specific antiserum

The Pla ORF was PCR-amplified from *Y. pestis* CO92 genomic DNA with primers 5′ Pla-SP_NcoI and 3′ Pla-SP_HindIII. The amplified ORF lacks the first 20 amino acids predicted by SignalP-6.0 (Teufel et al.) to encode the signal peptide of Pla (51). The amplicon was TA-cloned into pGEM-T Easy (Promega Corporation, Madison, WI) and confirmed by Sanger sequencing. The Pla ORF was then subcloned into pProEX-Htb (Thermo Scientific) via NcoI and HindIII restriction sites to produce a Pla fusion with an N-terminal His_6_ tag. To produce recombinant His_6_-Pla, BL21 (DE3) LOBSTR (Kerafast, Newark, CA) was transformed with pProEX-Htb::Pla. Cultures were induced in LB broth for 3 h with 1 mM IPTG at 37°C. Recombinant Pla was affinity-purified with HisPur Ni-NTA resin under non-native conditions per the manufacturer’s protocol. Fractions containing the recombinant Pla were combined and buffer exchanged to 2 M urea with phosphate-buffered saline (PBS) using an Amicon Ultra-15 mL 10 kDa MWCO centrifugal-filtration unit, and the final product was then resolubilized by adding 5% SDS. The concentration of the recombinant protein was calculated using the DC Protein Assay Kit (Bio-Rad Laboratories, Hercules, CA). Pla-specific antiserum was generated in rats as previously described with minor modifications (52). Briefly, 60 μg of recombinant Pla was electrophoresed by SDS-PAGE. The gel was stained with non-fixing Coomassie blue, and gel slices containing Pla were excised. Gel slices were ground in a sterile disposable mortar and pestle in PBS. The pulverized gel slices were then combined with an equal volume of AddaVax adjuvant (InvivoGen, San Diego, CA). Three- to four-week-old female Sprague-Dawley rats (Charles River Laboratories, Wilmington, MA) were injected subcutaneously at two sites with 200 μL of emulsion per injection (total of ~25 μg of recombinant Pla in each rat). Rats were boosted twice at 4-week intervals with ~25 μg of recombinant Pla and AddaVax mixture. Rats were euthanized, and the serum was collected 2 weeks after the second boost. Immunizations were performed in accordance with the *Guide for the Care and Use of Laboratory Animals*, the Public Health Science Policy on Humane Care and Use of Laboratory Animals, and the Animal Welfare Act, and the protocol used was approved by the University of Arkansas for Medical Sciences (UAMS) Institutional Animal Care and Use Committee. UAMS is accredited by the International Association for Assessment and Accreditation of Laboratory Animals Care.

### DNA binding by fluorescence polarization

Oligonucleotides were resuspended in nuclease-free H_2_O (Ambion). To anneal ssDNA oligos, a 50 μL solution containing 10μM of the 5′-FAM-labeled oligonucleotides for *gyrB*, *mgtB, pla*, and PhoP Mut BB *pla*, 15 μM of the unlabeled reverse complement oligonucleotides, 25 mM Tris-HCl, and 100 mM NaCl was used. Oligonucleotide solutions were heated to 95°C, incubated for 5 min, and allowed to cool for 1 h to reach ambient temperature. The annealed substrates were stored at room temperature and kept in the dark prior to analysis. All oligos measured 38 nt in length. The affinity of *Y. pestis* PhoP toward 5′-FAM-labeled *gyrB, mgtB, pla,* and *pla* containing a PhoP Mut BB was determined using fluorescence polarization on a plate reader (Biotek SynergyH4) at 37°C. Titrations of PhoP, ranging in concentration from 50 nm to 10 μM, were mixed with a fixed concentration of dsDNA substrates (1 nM) in 40 mM HEPES (pH 7.5) buffer containing 5 mM MgCl_2_, 2 mM β-mercaptoethanol (β-ME), 0.1 mg/mL bovine serum albumin, and 50 mM NaCl. The change in fluorescence polarization at every concentration of protein was measured, plotted as a function of protein concentration, and was fit to a quadratic equation, as described previously, to determine the values of equilibrium dissociation constants (53).

### PhoP/PhoQ induction

WT, Δ*phoP*, and Δ*phoP::phoP pgm^–^* CO92 *Y. pestis* were evaluated under a variety of conditions (50 μg/mL LL-37 [Tocris], 20 μg/mL human β-defensin 1 [Genscript], pH 6 BHI [±0.2], PMH minimal media [10 mM glucose] containing 20 mM MgCl_2_ [Complete] or 10 μM MgCl_2_ [Low Mg^+2^], and complete PMH minimal media containing 0.2% glycerol or pH 6 complete PMH minimal media containing 0.2% glycerol in place of glucose) to evaluate the effect of PhoP/PhoQ activation on *pla* expression (54, 55). Three separate liquid cultures of WT and Δ*phoP* CO92 *Y. pestis* were grown overnight at 37°C with constant shaking in 10 mL BHI broth containing 2.5 mM CaCl_2_. The following morning, each culture was subcultured to an OD_600_ of 0.02 in 10 mL BHI broth containing 2.5 mM CaCl_2_ (pH 6 BHI, 50 μg/mL LL-37, and 20 μg/mL human β-defensin 1) or 10 mL PMH complete minimal media containing 2.5 mM CaCl_2_ (Low Mg^+2^). Once cultures reached an OD_600_ of ~0.1, compounds were added to concentrations specified above, or the cultures were pelleted, washed once with 1× PBS, and resuspended in PMH minimal media containing 10 μM Mg^+2^ or pH 6 BHI broth. Prior to the addition of each treatment, 1 mL of each culture was pelleted and resuspended in 1 mL Trizol (Thermo) to serve as a 0-h/baseline sample. Following a 2-h incubation, the OD_600_ was determined for each culture, and 1 mL of each culture was pelleted and resuspended in 1 mL Trizol. All Trizol samples were placed at −80°C until processed for qRT-PCR.

### Quantitative reverse transcription PCR

Following resuspension in Trizol, RNA was isolated according to the manufacturer’s instructions. DNA was removed from each sample by DNase treatment (Turbo DNase Kit) according to the manufacturer’s instructions, and cDNA was generated using SuperScript IV First-Strand Complementary DNA Synthesis Kit (Thermo). qRT-PCR was performed using PowerUp SYBR Green Master Mix (Thermo) with a QuantStudio 6 system. qRT-PCRs were performed on MicroAmp Optical 96-well Reaction Plates (Thermo) using 1:100 cDNA dilutions in a total reaction volume of 15 μL. Fold changes were calculated using the ΔΔCT method, normalized to the *gyrB* gene. Primers used for qRT-PCR are included in Table 1.

**TABLE 1:** Primers and oligonucleotides used in this study

Primers/Oligos	Sequence (5′–3′)
5′ Pla-SP NcoI	CCATGGCATCATCTCAGTTAATACCAAATATAT
3′ Pla-SP HindIII	AAGCTTCAGAAGCGATATTGCAGACCCG
qRT *pla* F	CCGAAAGGAGTGCGGGTAAT
qRT *pla* R	GCAGCATCTCCGCCAATAGA
qRT *gyrB* F	ATCCGTTGTTAACGCCCTGTCTGA
qRT *gyrB* R	ACAGTAGTCCCGGTTTGCTCAGTT
Anisotropy *pla* (FAM-tagged)	TATCGATTATTGTTTAATATTTTTACATTATTAAAAAT
Anisotropy *pla* complement	ATTTTTAATAATGTAAAAATATTAAACAATAATCGATA
Anisotropy *mgtB* (FAM-tagged)	TCCTTTATTTTGTTTAAGTATTGTTTAAGAATATACCC
Anisotropy *mgtB* complement	GGGTATATTCTTAAACAATACTTAAACAAAATAAAGGA
Anisotropy *pla* Mut BB (FAM-tagged)	TATCGATTATCTTTTAATATTACCACGGTATTAAAAAT
Anisotropy *pla* Mut BB complement	ATTTTTAATACCGTGGTAATATTAAAAGATAATCGATA
Anisotropy *gyrB* (FAM-tagged)	TTTCAGGCAAAAAAAAGCCCAGTGGCAGTTTTCCCACC
Anisotropy *gyrB* complement	GGTGGGAAAACTGCCACTGGGCTTTTTTTTGCCTGAAA

### Western blot analysis and quantification

Following PhoP/PhoQ induction, samples were resuspended in lysis buffer (lysozyme [500 μg/mL], BugBuster [1:100 dilution], DNase [1 μg/mL], MgCl_2_ [5 mM], and protease inhibitor), normalized to 1 OD. Samples were vortexed vigorously for 15–20 s, left at RT for 15–20 min with occasional vortexing, and placed at −80°C. Prior to the addition of 4× protein loading dye, total protein concentration was calculated using a BCA Protein Assay Kit (Pierce). 5–25 μg of total protein was boiled for 5 min at 96°C, placed on ice, and briefly centrifuged to collect the sample. Samples were loaded onto a pre-cast 10% Mini-PROTEAN TGX gel (BioRad) and run at 180 V for 30–45 min. Samples were transferred to a PVDF membrane with a Trans-Blot Turbo System (BioRad) using the Mixed MW protocol. The membrane was washed in 1× TBS once for 5–10 min and then blocked with 1× TBST 5% non-fat milk (blocking solution) for 2 h at RT. The membrane was cut in half between 37 kDa and 50 kDa and incubated overnight in blocking solution containing α-Pla polyclonal antiserum at a 1:1,000 dilution or α-GroEL antibody (Abcam) at a 1:5,000–10,000 dilution at 4°C with gentle shaking. The membranes were washed with 1× TBST six times for 5–10 min per wash. The membranes were then incubated in blocking solution containing goat anti-rat horseradish peroxidase (HRP) or goat anti-rabbit HRP 2° Ab for 1 h at RT with gentle shaking and then washed with 1× TBST six times for 5–10 min per wash. Tween-20 was removed by washing with 1× TBS two or three times for 5 min per wash. Membranes were developed using either BioRad Clarity ECL Substrate or SuperSignal West Pico PLUS Chemiluminescent Substrate (Thermo Scientific) according to the manufacturer’s protocols. Membranes were imaged, and protein was quantified using ImageJ. For densitometry calculation using ImageJ, protein band selections were normalized to the largest band, and the mean gray value was analyzed for both the band and the image background. Measurements for each protein band and background were input into Microsoft Excel, inverted (255 – X), and a net value was calculated by deducting the inverted background values from the inverted band values. For each sample, the net protein/net loading control ratio was calculated, and this ratio was used to calculate the relative protein abundance of mutant and complement strains compared to WT bacteria.

### Statistical analysis

All statistical analyses were completed using an unpaired Student’s *t*-test with Welch’s correction or one-way ANOVA. All statistical analyses were performed using GraphPad Prism software.

## AFTER EPUB

*[This article was published on 23 December 2025 with missing Acknowledgements. The Acknowledgements section was added in the current version, posted on 8 January 2026.]*

## SUPPLEMENTAL MATERIAL

Figure S1 jb.00357-25-s0001.docx Densitometry analysis of Western blots of total *Pla* expression.

ASM does not own the copyrights to Supplemental Material that may be linked to, or accessed through, an article. The authors have granted ASM a non-exclusive, world-wide license to publish the Supplemental Material files. Please contact the corresponding author directly for reuse.
