# Supplementary material for: Yersinia pestis plasminogen activator protease is regulated by the PhoP/PhoQ two-component system
Source: J Bacteriol. 2025 Dec 23;208(1):e00357-25. doi: 10.1128/jb.00357-25 (PMC12826059; doi:10.1128/jb.00357-25)
Supplement: Figure S1 — Densitometry analysis of Western blots of total Pla expression. [file jb.00357-25-s0001.docx]

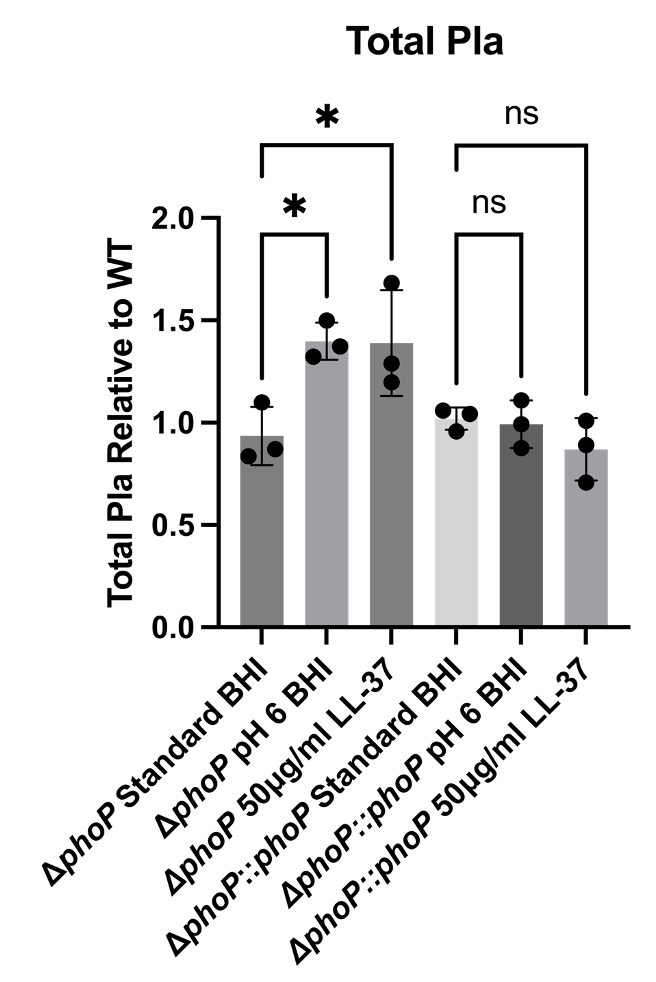


**Figure S1.** Densitometry analysis of average total Pla [α-Pla (35kDa), β-Pla (33kDa), and γ-Pla (31kDa)]/GroEL ratio from three different Western Blots of protein lysates from Δ*phoP* or Δ*phoP*::*phoP* strains relative to WT in Standard BHI, pH 6 BHI, or 50 μg/ml LL-37. Ratios ns, not significant; *, *P* ≤ 0.05 (One-Way ANOVA). N=3.
